# Genome-wide association study of milk and reproductive traits in dual-purpose Xinjiang Brown cattle

**DOI:** 10.1186/s12864-019-6224-x

**Published:** 2019-11-08

**Authors:** Jinghang Zhou, Liyuan Liu, Chunpeng James Chen, Menghua Zhang, Xin Lu, Zhiwu Zhang, Xixia Huang, Yuangang Shi

**Affiliations:** 1 School of Agriculture, Ningxia University, Yinchuan, China; 2 Department of Crop and Soil Sciences, Washington State University, Pullman, Washington USA; 3 College of Animal Science, Xinjiang Agricultural University, Urumqi, China

**Keywords:** Cattle, Dual-purpose, Milk, SCS, Reproduction, GWAS

## Abstract

**Background:**

Dual-purpose cattle are more adaptive to environmental challenges than single-purpose dairy or beef cattle. Balance among milk, reproductive, and mastitis resistance traits in breeding programs is therefore more critical for dual-purpose cattle to increase net income and maintain well-being. With dual-purpose Xinjiang Brown cattle adapted to the Xinjiang Region in northwestern China, we conducted genome-wide association studies (GWAS) to dissect the genetic architecture related to milk, reproductive, and mastitis resistance traits. Phenotypic data were collected for 2410 individuals measured during 1995–2017. By adding another 445 ancestors, a total of 2855 related individuals were used to derive estimated breeding values for all individuals, including the 2410 individuals with phenotypes. Among phenotyped individuals, we genotyped 403 cows with the Illumina 150 K Bovine BeadChip.

**Results:**

GWAS were conducted with the FarmCPU (Fixed and random model circulating probability unification) method. We identified 12 markers significantly associated with six of the 10 traits under the threshold of 5% after a Bonferroni multiple test correction. Seven of these SNPs were in QTL regions previously identified to be associated with related traits. One identified SNP, *BovineHD1600006691,* was significantly associated with both age at first service and age at first calving. This SNP directly overlapped a QTL previously reported to be associated with calving ease. Within 160 Kb upstream and downstream of each significant SNP identified, we speculated candidate genes based on functionality. Four of the SNPs were located within four candidate genes, including *CDH2*, which is linked to milk fat percentage, and *GABRG2,* which is associated with milk protein yield.

**Conclusions:**

These findings are beneficial not only for breeding through marker-assisted selection, but also for genome editing underlying the related traits to enhance the overall performance of dual-purpose cattle.

## Background

The Xinjiang Brown was recognized as a new dual-purpose cattle breed in China in 1983 [1]. Xinjiang Brown cattle have strong adaptability and resistance under extreme weather conditions. For example, these cattle can graze in temperatures below -40 °C and in snow up to 20 cm deep [1]. Because of these superior characteristics, the breed has spread widely across the northern area of Xinjiang. By the end of 2017, the population had reached nearly 1.5 million, including hybrid progeny [2]. Similar to breeders of other dual-purpose cattle breeds, Xinjiang Brown breeders took both dairy and beef traits into consideration to achieve comprehensive breeding objectives. Characteristics unique to dual-purpose cattle must be preserved, including the capacity to produce multiple products that can adapt to market demands. This product flexibility is particularly beneficial to small-scale herdsman who are more financially vulnerable to the whims of market changes and consumer preferences.

With the development of genotyping technologies and new genetic analysis methods, the genetic architecture of economically important traits have been explored across different cattle breeds and populations. Substantial genomic regions have been identified [3–6]. According to Release 36 in the Animal Quantitative Trait Loci (QTL) Database [7], 41,234 QTL are associated with 154 milk traits, 42,648 QTL with 71 reproductive traits, and 4081 QTL with 92 health traits. Potential candidate genes were also identified for these traits. For example, the *DGAT1* gene associates with milk composition and yield traits [8, 9] and has been validated as a major gene in Holstein populations across multiple countries [10]. *FASN* has a significant effect on milk fat component traits [11, 12]. *BRCA1* has an effect on somatic cell score (SCC), which influences mastitis disease in dairy cows [13, 14]. For reproductive traits, the *GH-L127 V* mutation was reported to be associated with calving interval in a Jersey cattle population [15].

Although many genome-wide association studies (GWAS) and genomic functional validation studies on dairy and beef cattle traits have been performed, few studies have focused on dual-purpose breeds and populations. For Xinjiang Brown, only a few genetic polymorphisms have been reported for milk composition, somatic cell score, and early growth traits [16–19]. Studies on the dual-purpose cattle breed, German Fleckvieh, reported a QTL on the Bos taurus (bovine) autosome (BTA) 5 associated with milk production [20] and two loci on BTA 14 and 21 associated with calving ease and growth-related traits [21]. Another study reported several SNPs associated with milk and functional traits in a population of the dual-purpose breed, Italian Simmental [22]. A few selection signature studies revealed several genetic variations in both dairy and beef cattle (Gir) populations [23, 24], and a few genetic polymorphism studies discussed the genetic architecture of milk production traits in the Italian Simmental breed [25, 26]. Despite the valuable information provided by these previous genomic studies, GWAS using high-density SNPs are still limited in dual-purpose breeds. Because the genetic linkage phase could be different across breeds and populations, using the previously identified markers to conduct marker-assisted selection is problematic, especially when marker density was low during the discoveries. Therefore, GWAS with high-density SNPs are needed to understand the genetic architecture of important, complex traits in dual-purpose cattle breeds.

In this study, we evaluated five milk production traits: milk yield (MY), fat yield (FY), protein yield (PY), fat percentage (FP), and protein percentage (PP); four reproductive traits: age at first service (AFS), age at first calving (AFC), gestation length (GL), and calving interval (CI); and one health trait: somatic cell score (SCS) in the Chinese dual-purpose cattle breed, Xinjiang Brown. We used milk production, reproductive, and health data records, collected during 1995–2017 on 2410 individuals, from four different breeding herds raised in the Xinjiang region of northwestern China. We used another 445 ancestors to obtain a total of 2855 individuals connected by pedigree to estimate variance components and breeding values. Ultimately, a total of 403 cattle were selected for genotyping with the 150 K Bovine BeadChip, which resulted in a total of 139,376 markers. Our objective was to identify SNPs associated with milk, reproductive, and health traits in the Xinjiang Brown for the benefit of marker-assisted selection and dissection of genetic architecture of these complex traits.

## Results

### Descriptive statistics

A total of 2410 individuals with 6811 reproductive records and 5441 milk records were used in this study. The descriptive statistics results of milk, health, and reproductive traits in Xinjiang Brown Cattle are shown in the Table 1. Based on the milk records, the mean 305-day milk yield (MY) was 4216.49 kg. This mean MY value is within the normal range compared with Chinese dual-purpose Sanhe cattle, Simental cattle, and Chinese Range Red cattle [27], but less than European dual-purpose Fleckvieh and Braunvieh breeds [26]. In our Xinjiang Brown population, mean milk fat percentage (FP) was 3.93%, similar to Fleckvieh and Braunvieh; mean milk protein percentage (PP) was 3.37%, higher than these two breeds [28]. The population’s mean milk fat yield (FY) and protein yield (PY) were 168.53 kg and 143.79 kg, respectively, which are both less than Fleckvieh and Braunvieh [28].

**Table 1:** Statistical description of study traits^a^

Traits	Mean	SD	Min	Max	h2	SE (h2)	Phenotypic Variance	Additive Variance	Residual Variance
Milk Traits									
MY (kg)	4126.49	1405.71	814	8444	0.40	0.017	17,027,917	6,811,167	10,216,750
FY (kg)	168.53	64.29	21.60	431.54	0.30	0.013	3123.71	937.11	2186.60
PY (kg)	143.70	51.42	24.23	302.72	0.20	0.011	1824.40	364.88	1459.52
FP (%)	3.93	0.83	2.04	7.00	0.08	0.009	0.68	0.05	0.63
PP (%)	3.37	0.38	2.16	6.13	0.30	0.014	0.14	0.04	0.10
Health Trait									
SCS	4.98	2.16	−2.05	10.95	0.08	0.008	4.29	0.34	3.95
Reproductive Traits									
AFS (days)	571.89	84.82	420.00	759.00	0.01	0.006	6814.98	68.15	6746.83
AFC (days)	877.65	87.85	616.00	1066.00	0.01	0.005	7400.67	66.79	7333.88
CI (days)	437.51	77.97	320.00	617.00	0.08	0.009	5615.80	449.26	5166.54
GL (days)	284.56	15.52	195.00	339.00	0.07	0.007	238.73	16.73	222.00

^a^*SD* Standard deviation, *h2* Heritability of traits, *SE* Standard error. Ten traits in the study are *MY* Milk yield, *FY* Fat yield, *PY* Protein yield, *FP* Fat percentage, *PP* Protein percentage, *SCS* Somatic cell score, *AFS* Age at first service, *AFC* Age at first calving, *CI* Calving interval, and *GL* Gestation length

Somatic cell score (SCS) was used as an indicator trait for udder health; the smaller the SCS, the lower the risk for mastitis [29]. SCS is not only important in dairy cattle, but is also crucial in dual-purpose breeds. In the study population, mean SCS was moderate, 4.98, with a heritability of 0.08.

Most reproductive traits are difficult to measure and vary across environmental conditions [30]. We selected age at first service (AFS), age at first calving (AFC), gestation length (GL), and calving interval (CI) because they are relatively easy to record and analyze. The averages were 571.89 days, 877.65 days, 437.51 days, and 284.56 days for AFS, AFC, GL, and CI, respectively. Heritabilities were low for all four traits, ranging from 0.01 to 0.08, which is consistent with findings from other studies on dairy and beef cattle [31, 32]. Together, these traits can reflect a cow’s production efficiency and body condition and are also important breeding objectives for the Xinjiang Brown.

### Phenotypic, genetic and residual correlation

The correlations and distributions of phenotypes, estimated breeding values (EBV), and residuals for each of the 10 study traits are shown in Additional file 1: Figure S1. The EBVs of all traits followed a normal distribution. We found strong correlations among MY, FY, and PY phenotypes, with correlation coefficients ranging from 0.78 to 0.92. The genetic correlation coefficients among EBVs were medium to high, ranging from 0.54 to 0.70. The correlation between MY and both FP and PP were negative and weak (genetic and phenotypic), which have also been reported in other studies [33]. Among the reproductive traits, the strongest phenotypic and genetic correlations were found between AFS and AFC, with correlation coefficients of 0.94 and 0.92, respectively. The smaller the AFS, the smaller the AFC. We were particularly interested in traits with high genetic correlations and focused on whether they shared common markers.

### Population stratification

**Fig. 1:**
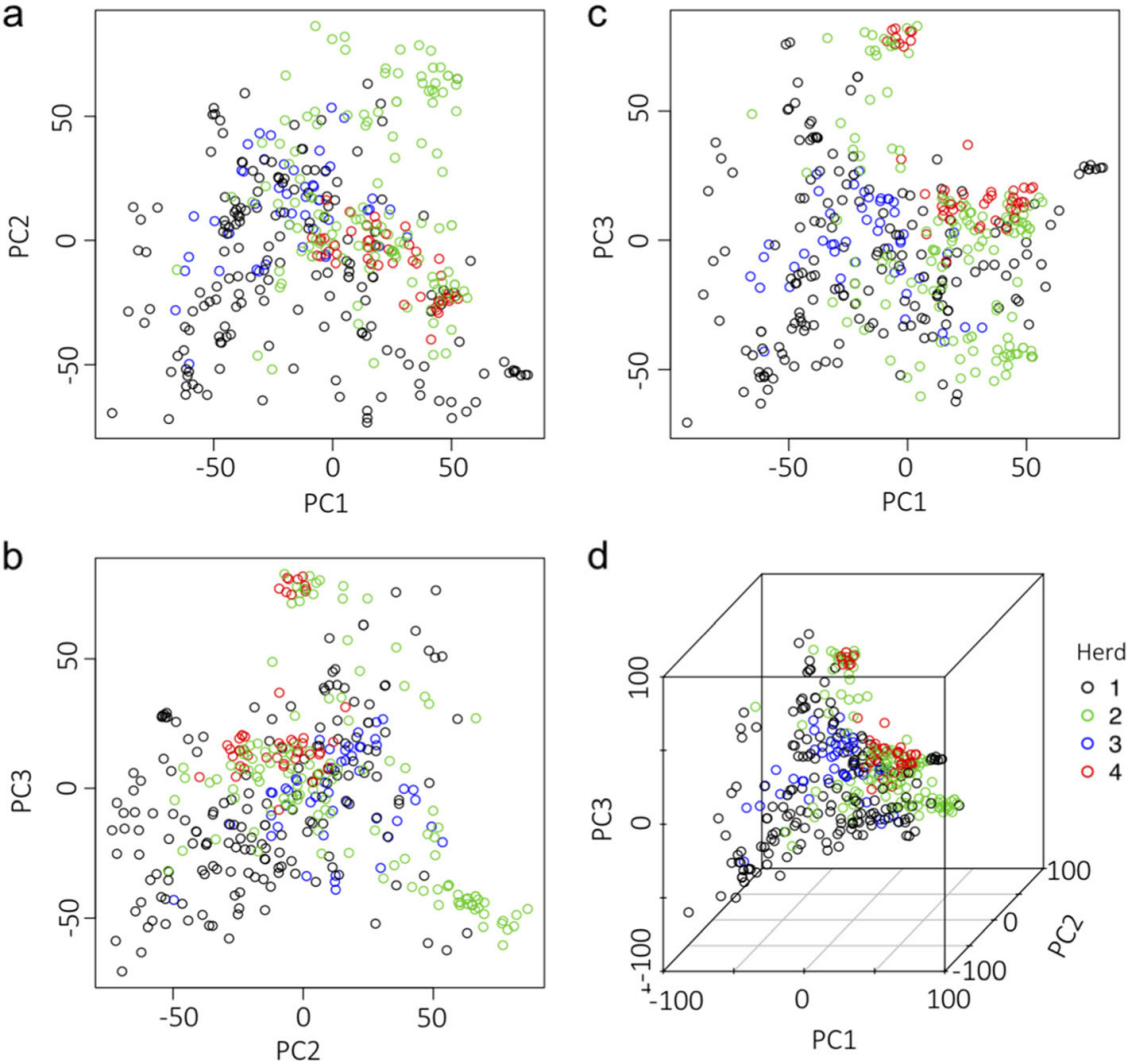
Population structure from the principal component analysis. A total of 11,8796 SNPs and 396 cattle were used to perform the principal component analysis. Population structure is shown as pairwise scatter plots (**a**, **b**, and **c**) and a 3D plot (**d**) of the first three principal components (PC) with colored circles that define the four herds. There are 173, 127, 48, and 48 cattle in herd 1, 2, 3, and 4, respectively

The PCA scatterplots illustrate a clear population structure for the 396 individuals in the four cattle herds that comprised our study population (Fig. 1). In the scatterplot of PC1 and PC2, the majority of cattle in herd 3 are completely separated from the majority of individuals in herd 4 (Fig. 1a). Similarly, most individuals from herd 1 and herd 2 split into another two distinct groups. Furthermore, several clusters of individuals, either from the same or from different herds, were observed in the scatterplot of PC1 and PC3 (Fig. 1b). Clusters of the same color represent closely related individuals from same herd. In contrast, we identified three distinct clusters of herd 2 (green) and herd 4 (red) individuals and two clusters of herd 2 (green) and herd 1 (black) individuals. These mixed clusters indicate that, although individuals may come from different herds, they still retain close genetic relationships. We further explored the relationships between the first three principal components (PCs) and the phenotypes of the 10 study traits with additional scatterplots (Additional file 4: Figure S4), but found no strong correlations.

### Genome-wide association studies

**Fig. 2:**
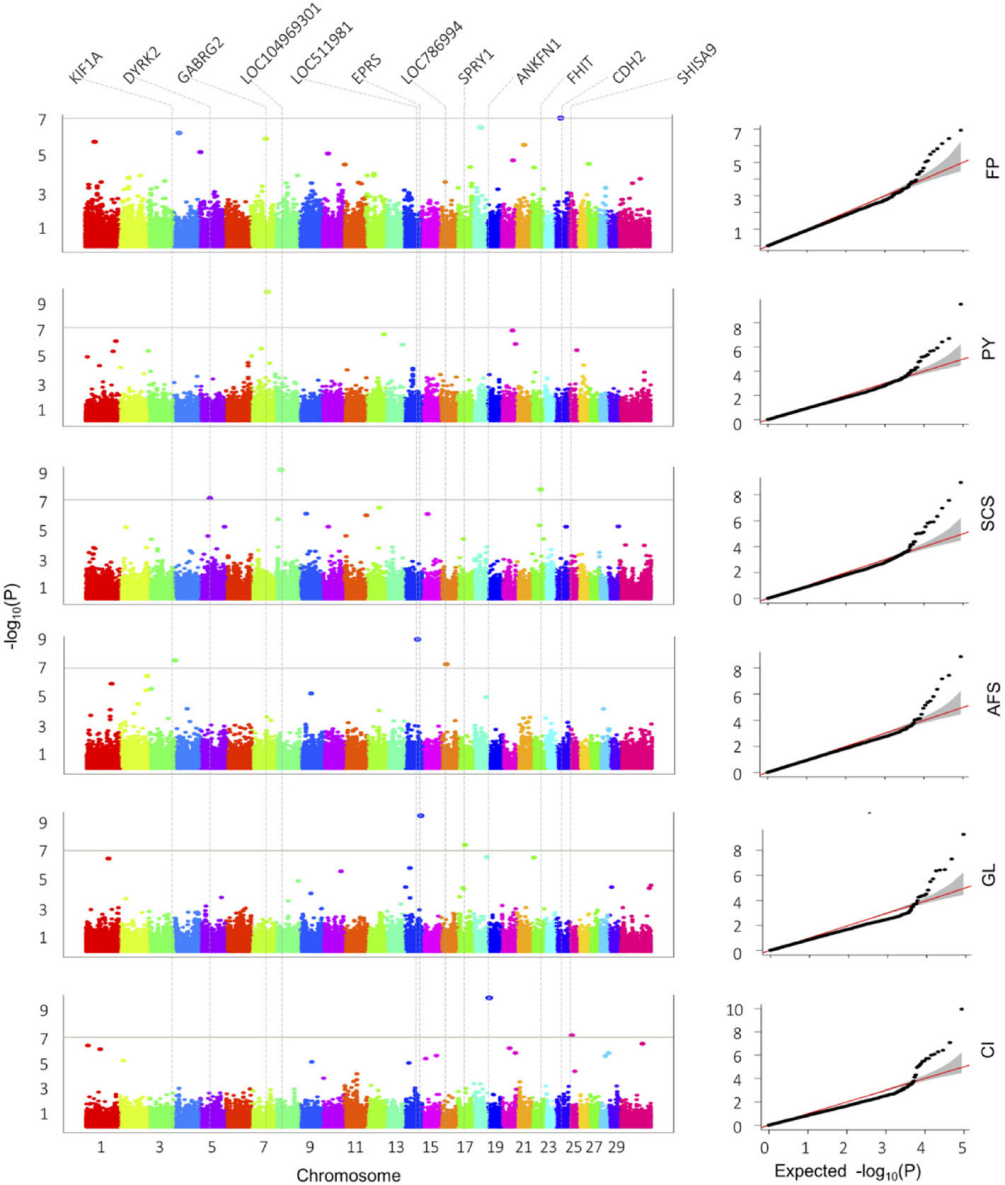
Manhattan and Q-Q plots of milk, reproductive, and health traits. FP = fat percentage, PY = protein yield, SCS = somatic cell score, AFS = age at first service, GL = gestation length, and CI = calving interval. The genome-wide association study was performed by FarmCPU software, with a significant *p*-value threshold set at *P* = 10–7. We identified the 12 nearest genes to each significant SNPs, which are labeled at the top of the Manhattan plot (left). Q-Q plots are displayed as scatter plots of observed and expected log *p*-values (right)

The FarmCPU method was used to perform the genome-wide association analysis. Because population structure can cause false positive results in GWAS, the first three PCs were added into our GWAS model. Ultimately, 12 SNPs passed the 5% threshold after a Bonferroni correction and were associated with six of the 10 study traits (Fig. 2). For milk traits, two significant SNPs were detected on Bos taurus autosome (BTA) 24 (*BovineHD2400007916*) and BTA 7 (*BTB-01731924*) and were associated with FP and PY, respectively. For the health trait, mastitis resistance, three significant SNPs were found on BTA 8 (*BovineHD0800007286*), BTA 22 (*BovineHD2200012261*), and BTA 5 (*BovineHD0500013296*) and were associated with SCS. For reproductive traits, three SNPs located on BTA 14 (*BovineHD1400016327*), BTA 3 (*BovineHD0300035237)* and BTA 16 (*BovineHD1600006691*) were significantly associated with AFS; two SNPs located on BTA 14 (*BovineHD1400021729*) and BTA 17 (*ARS-USMARC_528*) were significantly associated with GL; and two SNPs located on BTA 19 (*Bovine HD1900002007*) and BTA 25 (*BovineHD2500003462*) were significantly associated with CI. We found no significant markers associated with MY, FY, PP, or AFC (Additional file 5: Figure S5).

To check for overlaps among the SNPs significantly associated with milk, reproductive, or health traits, we created a heat map using different bin sizes and several significant *p* thresholds (Additional file 3: Figure S3). The visual effect of Additional file 3: Figure S3 is a combination of both the strength of signals and the bandwidth. For a small bin, the band is visible only when the signal is strong. For the same level of signals, a band becomes visible when it is wide enough. We found one overlapping SNP (*BovineHD1600006691*) at 24.2 Mb on BTA 16 that associated with both AFS and AFC. This SNP has also been reported in a QTL region associated with calving ease [34]. Additionally, most of the SNPs we identified have been previously located in QTL regions that are associated with traits related to our study traits. We mapped 12 candidate genes on 11 autosomes, based on the physical position of the significant SNPs (Fig. 2, Table 2). Four SNPs are within genes, including CDH2, which is linked to FP, and GABRG2, which is associated with PY. The other SNPs are within 156 kb or less of a gene.

**Table 2:** GWAS-identified significant SNPs, associated traits, and nearest candidate genes^a^

Trait	SNP	Chr.	Position (bp)	MAF	Nearest Gene	Distance (kb)	*P*-value
Milk Traits							
FP	BovineHD2400007916	24	29,095,464	0.370	CDH2	Within	1.19E-07
PY	BTB-01731924	7	75,830,763	0.140	GABRG2	Within	2.98E-10
Health Trait							
SCS	BovineHD0800007286	8	24,250,348	0.484	LOC104969301	121	1.13E-09
SCS	BovineHD2200012261	22	42,292,699	0.249	FHIT	159	2.61E-08
SCS	BovineHD0500013296	5	46,291,333	0.460	DYRK2	29	1.04E-07
Reproductive Traits							
AFS	BovineHD1400016327	14	58,781,799	0.378	LOC511981	69	1.32E-09
AFS	BovineHD0300035237	3	120,496,661	0.196	KIF1A	4	3.69E-08
AFS	BovineHD1600006691	16	24,235,446	0.063	EPRS	Within	6.76E-08
GL	BovineHD1400021729	14	77,464,140	0.370	LOC786994	77	5.15E-10
GL	ARS-USMARC-528	17	34,752,485	0.424	SPRY1	Within	4.99E-08
CI	BovineHD1900002007	19	7,557,250	0.278	ANKFN1	34	1.09E-10
CI	BovineHD2500003462	25	12,378,774	0.472	SHISA9	146	8.29E-08

^a^*SNP* Single nucleotide polymorphism, *MAF* Minor allele frequency, *Chr.* Chromosome, *FP* Fat percentage, *PY* Protein yield, *SCS* Somatic cell score, *AFS* Age at first service, *GL* Gestation length, *CI* Calving interval

## Discussion

### Population stratification

Population stratification is an important issue in population-based association studies [35, 36]. Because allele frequency may differ in sample individuals due to systematic ancestry differences [37], hidden population structure may cause spurious results and reduce the statistical power in GWAS. Consequently, stratification in the experimental population must be corrected [38–40]. In this study, our Xinjiang Brown experimental cattle were selected from four different commercial herds. Each year, foreign blood was introduced into each herd to improve population productivity, and sometimes cattle were transferred among herds. Thus, we hypothesized that some hidden structure should be inherent in our experimental population. Population structure is one of the major cause spurious association and must be accounted through stratified analyses such as genomic control, structured associations, and PCA [41]. We used PCA to detect the stratification and found a clear subpopulation structure (Fig. 1). For example, herd 3 and herd 4 exhibited an obvious clustering pattern and were completely separated by the first PC. Herd 2 and herd 4 exhibited an overlapping pattern, indicating that individuals from these two herds have a closer genetic relationship than individuals from other herds.

Cryptic relationships among individuals is another major source of spurious associations. Several methods have been developed to correct both population stratification and cryptic relationships to screen markers across genomes. Ideally, a one-step approach would perform the best by optimization over population structure, cryptic relationships, and genetic markers simultaneously; however, the associated computational burden prevents full optimization for practical uses. Furthermore, robust approximation was achieved with a dramatic reduction in computing time. For example, the EMMAx and P3D algorithms deliver almost identical results for full optimization of genetic and residual variance estimates for every testing marker, using the fixed and random effects mixed linear model (MLM).

The computing time of the MLM was further improved by splitting the model into a fixed effect model and a random effect model. The fixed effect model is used for testing markers, one at a time. The random effect model is used to select markers that are used as covariates in the fixed effect model. The fixed effect model and the random effect model are used iteratively until no change occurs in the covariates. Compared to the kinship based on all the available markers, the kinship based on the selected markers has the best likelihood for the specific trait of interest. This method was named the Fixed and random model Circulating Probability Unification (FarmCPU). Both simulation and analyses on real traits demonstrated that FarmCPU has higher statistical power than the regular mixed method using all available markers to build kinship.

Given this population stratification, we used two models to perform GWAS using FarmCPU, with and without the first three PCs as covariates. Without including the PCs, we found 20 significant markers associated with eight of the 10 traits (Additional file 6: Figure S6). After including the PCs, 18 of these 20 significant markers disappeared and 10 new SNPs surfaced. We calculated the inflation factor to check whether significant population structure remained (Additional file 7: Table S1). The result showed minimal inflation using FarmCPU. Both quantile-quantile plots (Q-Q plot) and the inflation factor exhibited the same trend. In fact, FarmCPU is conservative, which even led to minor deflation. Because the previous study [42] suggested including PCs to ensure population structure is incorporated when performing FarmCPU, we used the model with PCs fitted as covariates. In total, the combined SNP-PCA model identified 12 significant markers associated with six of the 10 traits (Fig. 2).

### Comparison of GWAS results

We found 12 significant markers associated with six important, complex traits in Xinjiang Brown cattle, based on a high-density SNP chip. Among them, two SNPs overlapped in both the SNP model and the combined SNP-PCA model. One SNP is seated on BTA 8 and significantly associated with SCS; the other SNP is on BTA 16 and significantly associated with AFS. Four SNPs were significantly associated with MY, FY, PP, and AFC when we used the SNP model, but these SNPs failed to pass the 5% threshold after a Bonferroni correction in the combined SNP-PCA model. Still, SNPs associated with FY (*Bovine HD1600007977*), PP (*Bovine HD2300015096*), and AFC (*Bovine HD1600006691*) are the most significant SNPs in both models. Our study is the first GWAS on milk, reproductive, and mastitis resistance traits in the Xinjiang Brown dual-purpose cattle breed. Only a limited number of studies have reported on similar traits in other dual-purpose breeds [20–26]; therefore, we compared our results with studies of single-purpose dairy and beef cattle breeds.

Milk composition traits are important breeding traits in both dairy and dual-purpose cattle breeds, especially in modern animal husbandry environments. We found two highly significant SNPs associated with milk composition traits. One SNP is associated with FP and is positioned within the cadherin-2 (*CDH2*) gene at 29.1 Mbp on BTA 24. *CDH2* is a protein encoding gene and participates in adipogenesis [43]. Knocking down *CDH2* to block the epithelial-mesenchymal transition-like response could weaken adipocyte lineage commitment [44]. Several previous studies have reported QTL near this SNP. For example, one study found a QTL region spanning 18.1–21.8 Mbp on BTA 24 that was associated with FP in a Danish Holstein population [45]. Another study mapped a QTL at 33.4 Mbp on BTA 24 that was associated with FP in another Holstein cattle population [46]. Furthermore, the cattle QTL database [7] reports an additional 14 QTL on either side of the FP-associated SNP we identified. These 14 QTL are associated with health, production, reproductive, and meat and carcass traits. One of the QTL that spans 21.8–31.0 Mbp on BTA 24 is significantly associated with SCS in Danish Holstein [47].

The other milk-related SNP we identified was significantly associated with PY and mapped at 75.8 Mbp on BTA 7, which is within a gene named Gamma-aminobutyric Acid Type A Receptor Gamma2 Subunit (*GABRG2*). *GABRG2* primarily contributes to gamma-aminobutyric acid (*GABA*)-gated chloride ion channel activity and participates in *GABA-A* receptor activity [48] and has been studied mostly in association with human idiopathic epilepsy [49, 50]. Among cattle genomic studies, a potential supporting study reported a nearby QTL region spanning 71.9–73.8 Mbp on BTA7 that was associated with PY in a US Holstein population [51]. Additionally, we found six other QTL in the cattle QTL database [7] that contained the PY-associated SNP we identified. Three of these QTLs are associated with milk FY in Holstein and Jersey cow populations [52]. One QTL is significantly associated with meat fat content in Nellore beef cattle [53]. Another QTL is linked to cold tolerance in a crossed beef cattle population [54]. And, the sixth one is linked to meat tenderness traits in five taurine cattle breeds [55].

SCS is highly correlated with mastitis in cattle populations [56, 57] and is usually selected as an indicator trait to reflect udder health status and mastitis resistance [58]. In this study, we mapped three highly significant, SCS-associated SNPs on BTA 5 (46.3 Mbp), BTA 22 (42.3 Mbp), and BTA 8 (24.2 Mbp). Three candidate genes were found nearby these three SNPs. One of the genes, named Dual Specificity Tyrosine Phosphorylation Regulated Kinase 2 (*DYRK2*), was reported to be related to udder support score trait in crossbred Bos indicus-Bos taurus cows [59]. Many QTL been reported for SCS. For example, a peak QTL region was found at 28.2–44.5 Mbp on BTA 5 in one Holstein population [60]. And, in another Holstein population, several QTL were found on BTA 22 within 1 Mbp of our identified SNP [51]. Two separate studies, performed in different years, reported the same QTL at 24.8 Mbp on BTA 8 that was related to SCS in Norwegian Red [61] and Red Pied dairy cattle [62]. The position of this QTL is close to the SNP we found on the same chromosome. We also found other studies that identified QTL regions associated with traits related to SCS and also contained the SCS-associated SNPs we identified in this study.

Before reproductive traits became important breeding objectives, most breeders focused on production traits [26]. However, to maintain balanced breeding, fertility traits have gained more and more attention in breeding schemes. Understanding the genetic architecture of low heritability traits, such as fertility traits, helps improve selection; thus, many GWAS on fertility traits have been performed [63–67]. In our GWAS, we found three highly significant SNPs associated with AFS. The first SNP is mapped at 120.4 Mbp on BTA 3; the nearby gene is Kinesin Family Member 1A (*KFM1A*). The second SNP is seated at 58.7 Mbp on BTA 14; the closest gene is a pseudo gene LOC511981. The third SNP is located at 24.2 Mbp on BTA 16 and within the Glutamyl-prolyl-tRNA Synthetase (*EPRS*) gene. Several QTL on BTA 16 contain the AFS-associated SNP we found. One of these QTL was previously reported to be related to calving ease in US Holstein cattle [51]; the other QTLs were related to weaning weight in Blonde d’Aquitaine beef cattle [68], birth weight in Angus beef cattle [69], and hip height in Qinchuan and Jiaxian Red beef cattle [70]. Both calving ease and body size traits are highly correlated with AFS.

For GL, we found two significant SNPs, one mapped at 77.5 Mbp on BTA 14 and the other mapped at 34.8 Mbp within the Sprouty RTK Signaling Antagonist 1(*SPRY1*) gene on BTA 17. The two SNPs we found significantly associated with CI were located at 7.6 Mbp on BTA 19 and at 12.4 Mbp on BTA 25. The nearest genes to these SNPs are Ankyrin-repeat and Fibronectin Type III Domain Containing 1 (*ANKFN1*) on BTA 19 and Shisa Family Member 9 (*SHISA9*) on BTA 25. A previously reported QTL region at 6.3–13.8 Mbp on BTA 25 was found to affect dystocia in a dairy population [65]. Another study reported a QTL at 6.3–17.7 Mbp on BTA 25 linked to no-return rate in Danish and Sweden Holstein cattle [66]. Both dystocia and no-return rate are fertility traits and, thus, related to the reproductive traits we studied.

## Conclusion

This study used a high-density SNP chip to perform GWAS with milk, reproductive, and mastitis traits in the Chinese dual-purpose cattle breed, Xinjiang Brown. We found 12 significant SNPs associated with six of the 10 traits studied. Seven of these SNPs overlap with QTL regions previously reported in studies of other cattle populations. The candidate gene, *CDH2*, participates in adipogenesis and may affect milk fat production. These results enhance our understanding of important, complex traits in the dual-purpose Xinjiang Brown cattle breed and contribute to further studies on validation of gene function and genomic selection.

## Methods

### Animals and phenotyping

Phenotypic data used in this study were collected during 1995–2017 from 2410 Xinjiang Brown cow individuals from four different breeding herds, they are Tacheng Area Xinjiang Brown Cattle Breeding Farm, Yili Xinhe Xinjiang Brown Cattle Breeding Farm, Urumqi Xinjiang Brown Cattle Breeding Farm, and the Xinjiang Tianshan Animal Husbandry and Bio-engineering Co., Ltd., located in Tacheng city, Yining city, Urumqi city and Changji city, respectively. Blood sample were collected from the coccygeal vine of the tail-head of cows by the Vacuum Blood Collector, cleaned the area before sampling and pressed the sample wound for a while to let it recover after extraction. The tail-head blood collection method we took is very quick, lower stress and almost painless for the cattle. We used an additional 445 ancestors, for a total of 2855 individuals connected by pedigree, to estimate the breeding values of five milk traits, four reproductive traits, and one health trait (Additional file 1: Figure S1, Additional file 2: Figure S2). Milk traits included milk yield (MY), fat yield (FY), protein yield (PY), fat percentage (FP), and protein percentage (PP). Reproductive traits were age at first service (AFS), age at first calving (AFC), gestation length (GL) and calving interval (CI). And, the health trait was somatic cell score (SCS).

### Genotyping and quality control

**Fig. 3:**
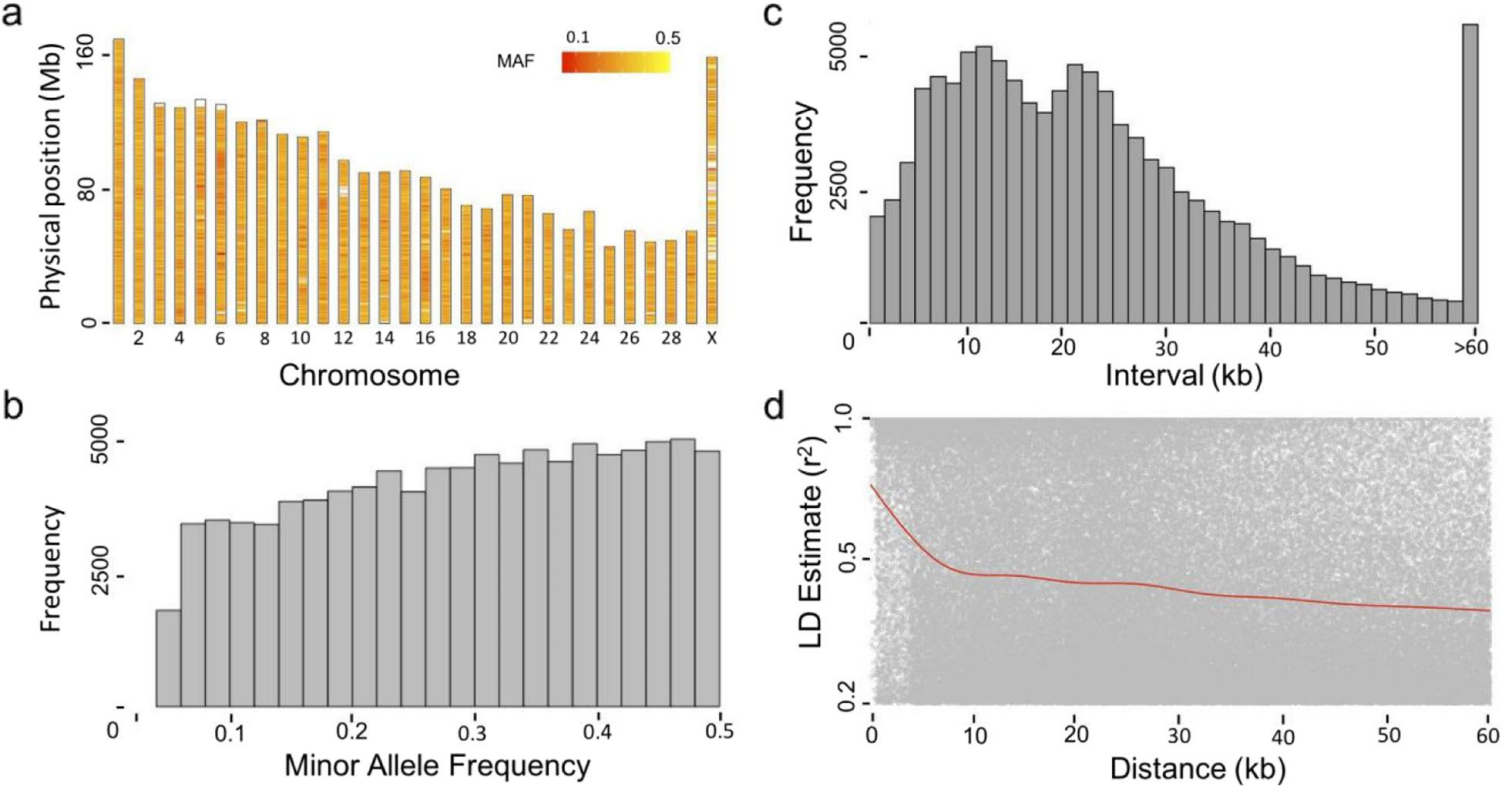
Properties of single nucleotide polymorphisms (SNPs). In total, 403 Xinjiang Brown individuals were genotyped by the Illumina GGP 150 k beadchip; 118,796 SNPs and 396 cattle passed filters and quality control. Marker distributions are displayed as the heatmap on 30 chromosomes by minor allele frequency (MAF) (**a**). MAF was re-calculated after quality control. Therefore, some SNPs remain with MAFs larther than 0.05, as shown by the histogram (**b**). Marker density is displayed by histogram according to the interval of adjacent SNPs (**c**). LD decay is shown by scatter plot according to pairwise distance and trend as a red line (**d**)

In total, 403 female cattle were selected for genotyping by using the Illumina 150 K Bovine BeadChip. Quality control was conducted by using Plink software [71] with criteria as follows: (1) individual call rate > 95%; (2) genotype call rate > 90%; (3) Hardy-Weinberg equilibrium *p*-value >1e-6; and (4) minor allele frequency (MAF) > 0.05. After quality control, 396 cows and 139,376 markers remained. The genotypes in A/T/G/C format were converted to numeric genotypes by iPat software [72]. The distribution of SNPs on each chromosome was relatively uniform, although a few chromosomes contained relatively large blank areas, especially chromosome X (Fig. 3). Chromosome 1 contained the greatest number of SNPs, whereas chromosome 25 contained the fewest. The distribution of MAF revealed that the MAF frequency increased with MAF, suggesting that SNPs on the Illumina 150 K Bovine BeadChip were selected for common SNPs. Xinjiang Brown had the same property as other breeds used for developing the chip. LD decay fell off quickly within 10 kb physical distance and then decreased slowly afterwards.

### Principal component analysis

The experimental Xinjiang Brown population came from four breeding herds. We used the Prcomp function in R to perform a principal component analysis (PCA). The PCA showed a clear population structure (Fig. 1). PC 1 showed the separation between the individuals of herd 3 (blue) and 4 (red). Some individuals from herd 4 and herd 2 (green) exhibited close relationships. Most individuals from herd 1 (black) clustered far away from the other herds.

### Estimated breeding values

Genetic analysis was carried out using DMU [73] software with the animal model as follows:

$$ {y}_{ijklm}=u+{Herd}_i+{Year}_j+{Season}_k+{Parity}_l+{a}_m+{e}_{ijklm,} $$

where *y*_*ijklm*_ is the phenotype in the *j*^*th*^ year, *k*^*th*^ season, and *l*^*th*^ parity of the *m*^*th*^ individual from *i*^*th*^ herd; *u* is overall mean of population, *Herd*_*i*_ is the herd effect according to a cow’s origin from one of the four herds; *Year*_*j*_ is the *j*^*th*^ year effect, *Season*_*k*_ is the *k*^*th*^ season effect, and Parity is the effect of *l*^*th*^ parity; *a* is the additive effect of *m*^*th*^ individual and *e* is the residual in the *j*^*th*^ year, *k*^*th*^ season, and *l*^*th*^ parity of the *m*^*th*^ individual from *i*^*th*^ herd. All effects were treated as random except the overall mean.

### Genome-wide association studies

The fixed and random model circulating probability unification (FarmCPU) method was used to carry out the genome-wide association analysis in this study [42]. The method uses a fixed effect model and a random effect model iteratively. The fixed effect model tests SNPs one at a time. The significant SNPs are evaluated in the random effect model and the validated SNPs are fitted as covariates in the fixed effect model to control population structure. These SNPs are selected based on the likelihood of using them to build the cryptic relationships among individuals. The iteration stops when no validated SNPs can be added as covariates. Both real data and simulated data has demonstrated that FarmCPU has higher statistical power than other methods, including the random effect model with kinship derived from all the markers, to conduct association tests [42].

## Supplementary information

**Additional file 1: Figure S1.** Correlations and distributions of phenotypes, EBVs (estimated breeding values), and residuals. The histograms on the diagonal are the distributions for each trait: MY = milk yield, FY = fat yield, PY = protein yield, FP = fat percentage, PP = protein percentage, SCS = somatic cell score, AFS = age at first service, AFC = age of first calving, GL = gestation length, and CI = calving interval. The upper triangle is comprised of the correlation coefficients among traits. The lower triangle is comprised of the pairwise scatter plots. Graphs a, b, c illustrate milk trait phenotypes, EBVs, and residuals, respectively. Graphs d, e, f illustrate reproduction trait phenotypes, EBVs, and residuals, respectively.

**Additional file 2: Figure S2.** Heatmap of milk and reproductive traits. Individuals are sorted row wise and traits column wise based on their similarity. The trait values were standardized and illustrated as heat map with red indicating highest and yellow the lowest. MY = milk yield, FY = fat yield, PY = protein yield, FP = the fat percentage, PP = protein percentage, SCS = somatic cell score, AFS = age at first service, AFC = age of first calving, GL = gestation length, and CI = calving interval.

**Additional file 3: Figure S3.** Display of significant markers as visible bands at different width. The number significant markers were determined by the *P*-value cut off with three levels illustrated on the left. The significant markers are displayed as bands with width indicated on the top starting from 100 kb to 10,000 kb. More bands are visible with wider bands than narrow bands. Wide band and less stringent *P* value threshold (e.g. to right) demonstrate pleiotropy of significant markers across traits. These traits include milk yield (MY), fat yield (FY), protein yield (PY), fat percentage (FP), protein percentage (PP), and somatic cell score (SCS). Reproductive traits include age at first service (AFS), age at first calving (AFC), gestation length (GL), and calving interval (CI).

**Additional file 4: Figure S4.** Scatter plot between principal components and trait phenotypes. We used these plot to determine which traits were correlated with population structure, represented by principal components (PC). Columns represent the first three principal components, rows represent each trait. Milk traits include milk yield (MY), fat yield (FY), protein yield (PY), fat percentage (FP), protein percentage (PP). and somatic cell score (SCS). Reproductive traits include age at first service (AFS), age at first calving (AFC), gestation length (GL), and calving interval (CI).

**Additional file 5: Figure S5.** Manhattan and Q-Q plots of non-significant GWAS results. GWAS was performed with FarmCPU software and a significant *p*-value threshold set at *P* = 10–7. Four of the 10 traits studied, milk yield (MY), fat yield (FY), protein percentage (PP), and age at first calving (AFC), resulted in no SNPs passing the Bonferroni threshold, as illustrated by the Manhattan plots on the left. On the right, Q-Q plots are displayed as scatter plots of true and expected log *p*-values.

**Additional file 6: Figure S6.** Manhattan and Q-Q plots of GWAS results, without considering population structure. These Manhattan plots (left) illustrate results from an association analysis model that did not consider population structure. GWAS was performed with FarmCPU software and a significant *p*-value threshold set at *P* = 10–7. Q-Q plots (right) are displayed as scatter plots of true and expected log *p*-values. Milk traits include milk yield (MY), fat yield (FY), protein yield (PY), fat percentage (FP), protein percentage (PP), and somatic cell score (SCS). Reproductive traits include age at first service (AFS), age at first calving (AFC), gestation length (GL), and calving interval (CI).

**Additional file 7: Table S1.** Genomic inflation factor (lambda) of each trait.

## Data Availability

The data and material used in this research are available from the corresponding author on request.

## Abbreviations

BTA: Bos Taurus autosome
EBV: Estimated breeding value
FarmCPU: Fixed and random model circulating probability unification
GWAS: Genome-wide association study
PCA: Principal component analysis
QTL: Quantitative trait loci
SNP: Single nucleotide polymorphism
