# Supplementary figures and images for: Genome-wide association study of milk and reproductive traits in dual-purpose Xinjiang Brown cattle
Source: BMC Genomics. 2019 Nov 8;20:827. doi: 10.1186/s12864-019-6224-x (PMC6842163; doi:10.1186/s12864-019-6224-x)

**
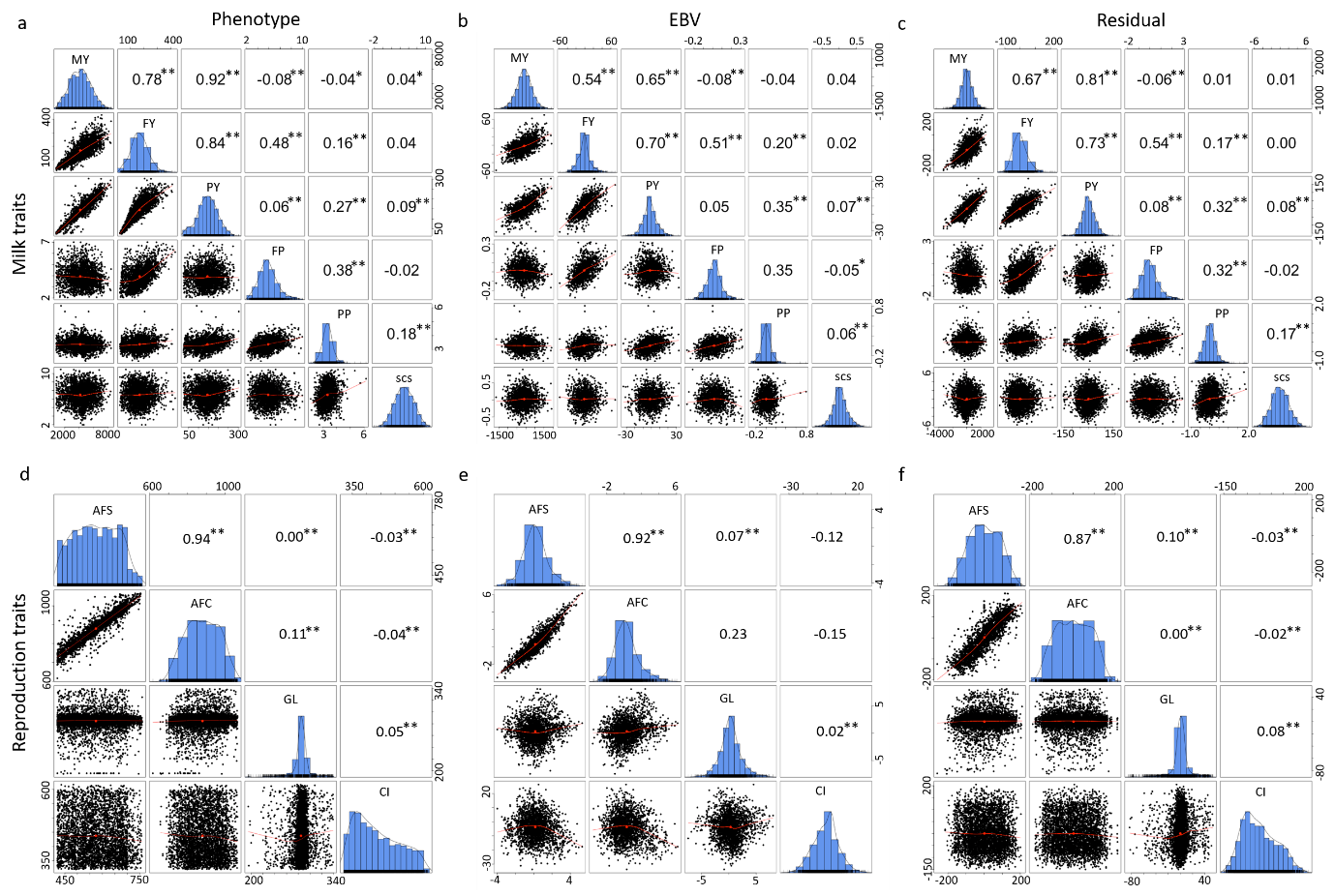
**

Supplement: Supplementary file 1 — Additional file 1: Figure S1. Correlations and distributions of phenotypes, EBVs (estimated breeding values), and residuals. The histograms on the diagonal are the distributions for each trait: MY = milk yield, FY = fat yield, PY = protein yield, FP = fat percentage, PP = protein percentage, SCS = somatic cell score, AFS = age at first service, AFC = age of first calving, GL = gestation length, and CI = calving interval. The upper triangle is comprised of the correlation coefficients among traits. The lower triangle is comprised of the pairwise scatter plots. Graphs a, b, c illustrate milk trait phenotypes, EBVs, and residuals, respectively. Graphs d, e, f illustrate reproduction trait phenotypes, EBVs, and residuals, respectively. [file 12864_2019_6224_MOESM1_ESM.docx]

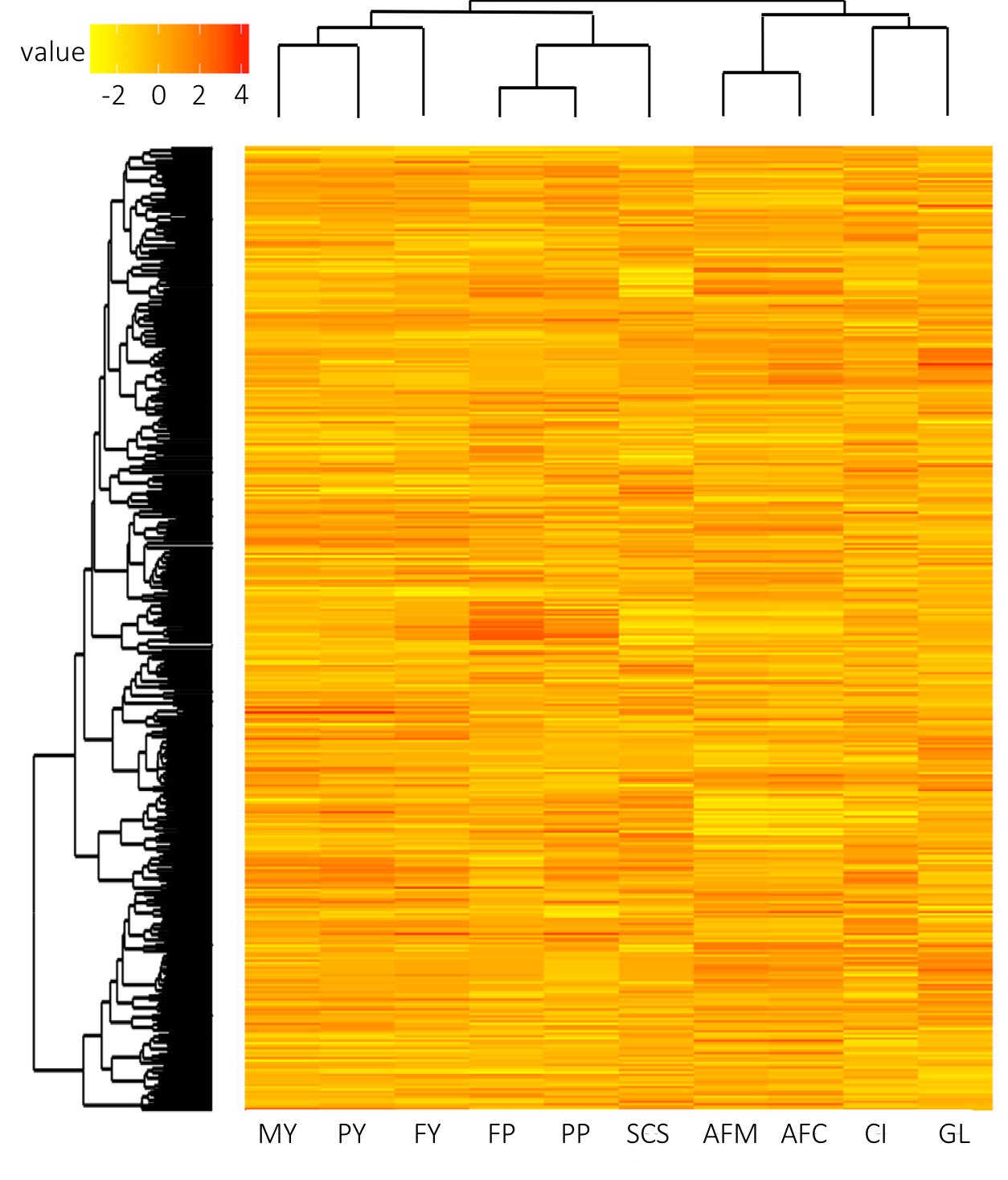

Supplement: Supplementary file 2 — Additional file 2: Figure S2. Heatmap of milk and reproductive traits. Individuals are sorted row wise and traits column wise based on their similarity. The trait values were standardized and illustrated as heat map with red indicating highest and yellow the lowest. MY = milk yield, FY = fat yield, PY = protein yield, FP = the fat percentage, PP = protein percentage, SCS = somatic cell score, AFS = age at first service, AFC = age of first calving, GL = gestation length, and CI = calving interval. [file 12864_2019_6224_MOESM2_ESM.docx]

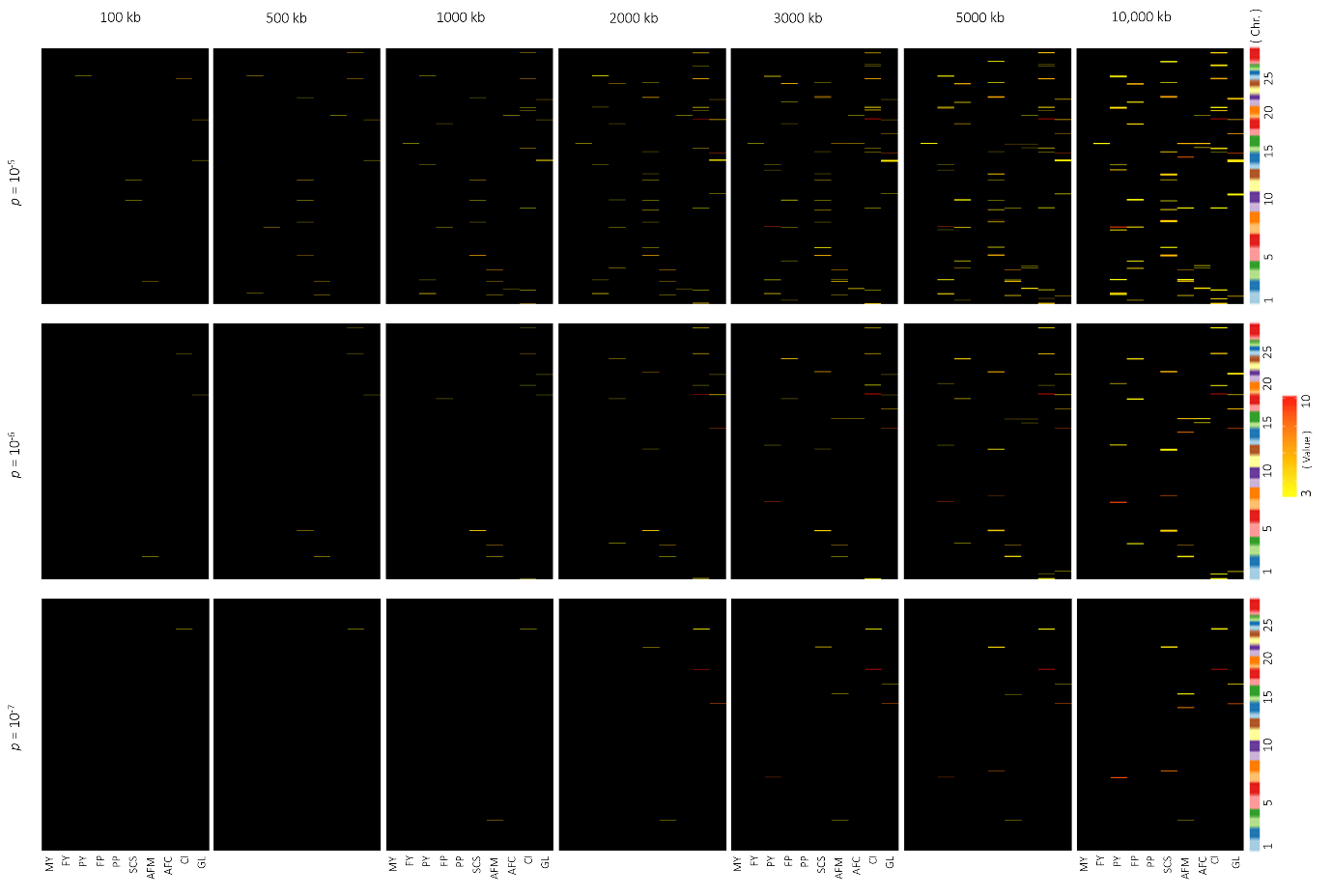

Supplement: Supplementary file 3 — Additional file 3: Figure S3. Display of significant markers as visible bands at different width. The number significant markers were determined by the P-value cut off with three levels illustrated on the left. The significant markers are displayed as bands with width indicated on the top starting from 100 kb to 10,000 kb. More bands are visible with wider bands than narrow bands. Wide band and less stringent P value threshold (e.g. to right) demonstrate pleiotropy of significant markers across traits. These traits include milk yield (MY), fat yield (FY), protein yield (PY), fat percentage (FP), protein percentage (PP), and somatic cell score (SCS). Reproductive traits include age at first service (AFS), age at first calving (AFC), gestation length (GL), and calving interval (CI). [file 12864_2019_6224_MOESM3_ESM.docx]

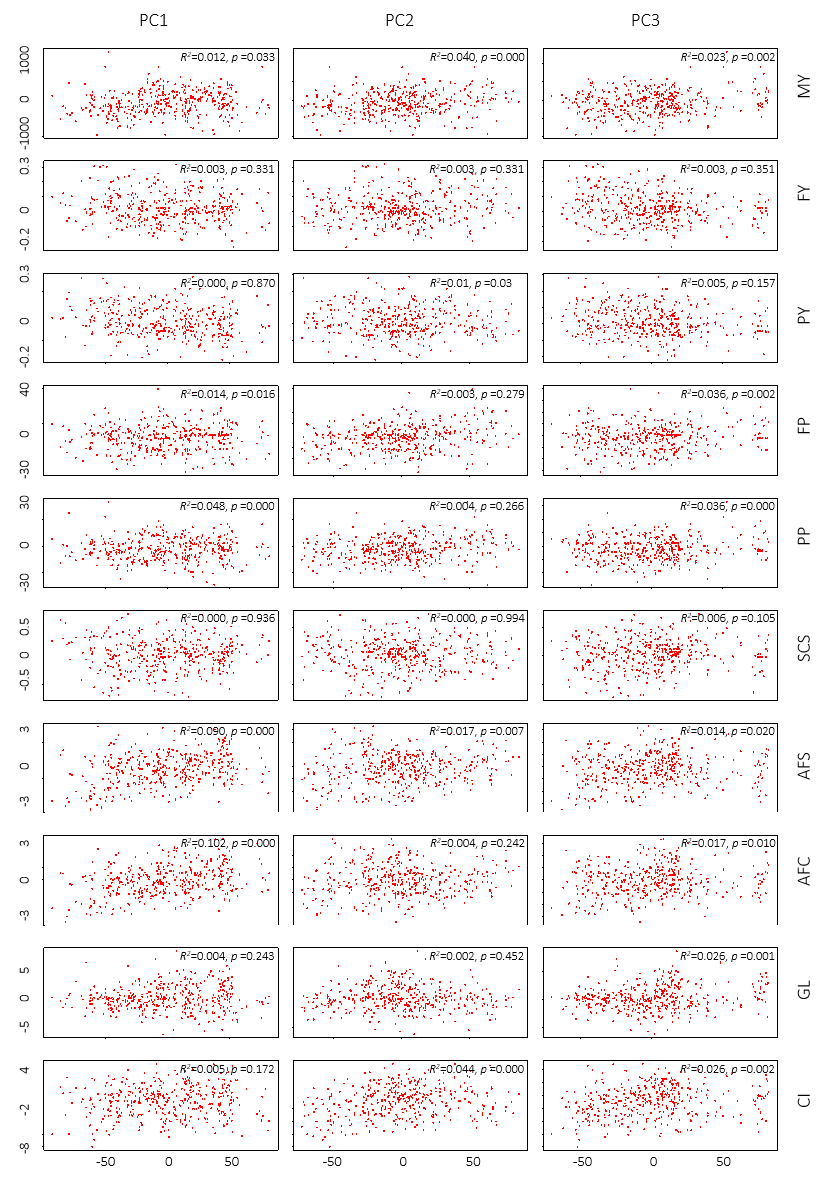

Supplement: Supplementary file 4 — Additional file 4: Figure S4. Scatter plot between principal components and trait phenotypes. We used these plot to determine which traits were correlated with population structure, represented by principal components (PC). Columns represent the first three principal components, rows represent each trait. Milk traits include milk yield (MY), fat yield (FY), protein yield (PY), fat percentage (FP), protein percentage (PP). and somatic cell score (SCS). Reproductive traits include age at first service (AFS), age at first calving (AFC), gestation length (GL), and calving interval (CI). [file 12864_2019_6224_MOESM4_ESM.docx]

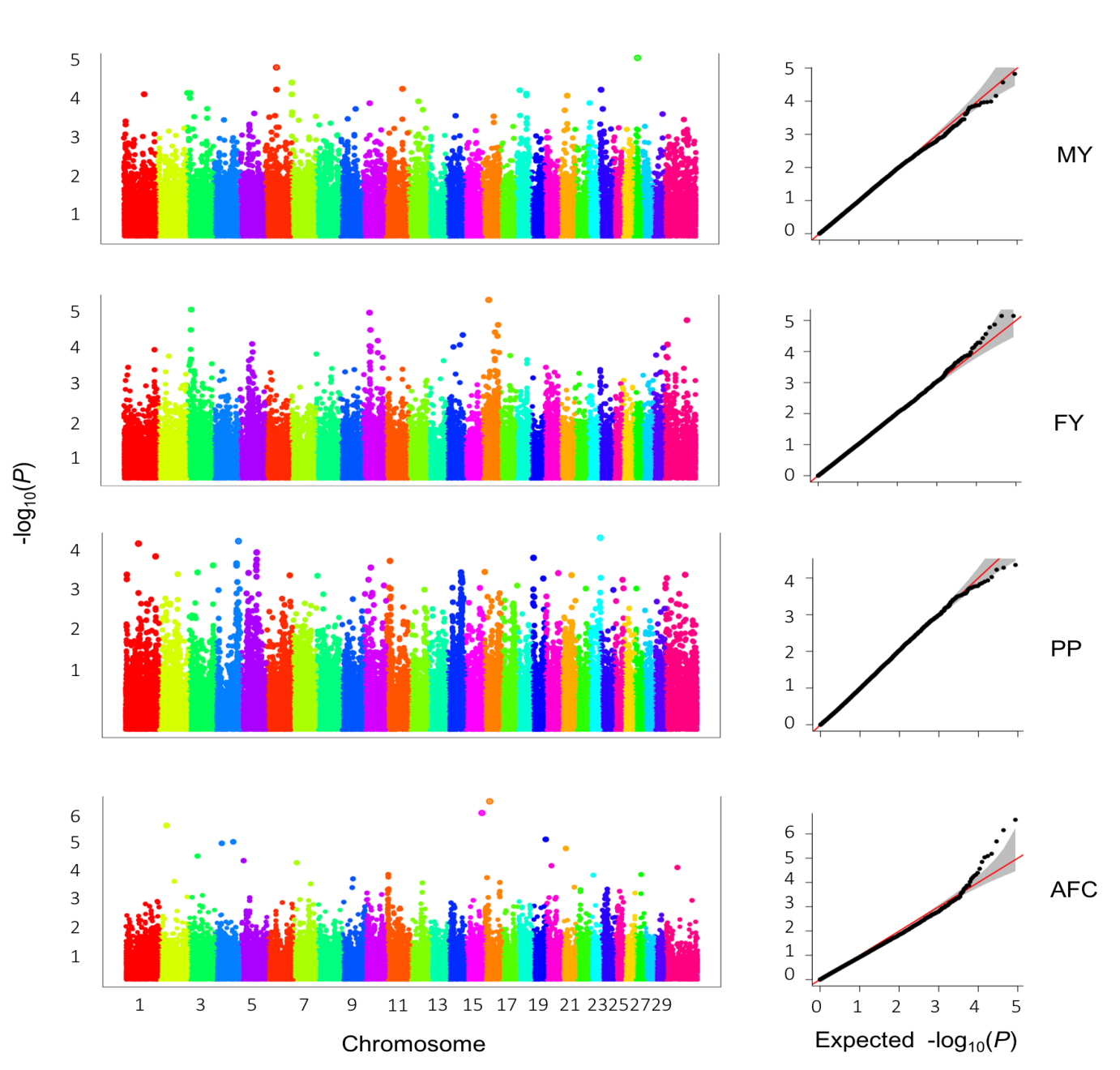

Supplement: Supplementary file 5 — Additional file 5: Figure S5. Manhattan and Q-Q plots of non-significant GWAS results. GWAS was performed with FarmCPU software and a significant p-value threshold set at P = 10–7. Four of the 10 traits studied, milk yield (MY), fat yield (FY), protein percentage (PP), and age at first calving (AFC), resulted in no SNPs passing the Bonferroni threshold, as illustrated by the Manhattan plots on the left. On the right, Q-Q plots are displayed as scatter plots of true and expected log p-values. [file 12864_2019_6224_MOESM5_ESM.docx]

**
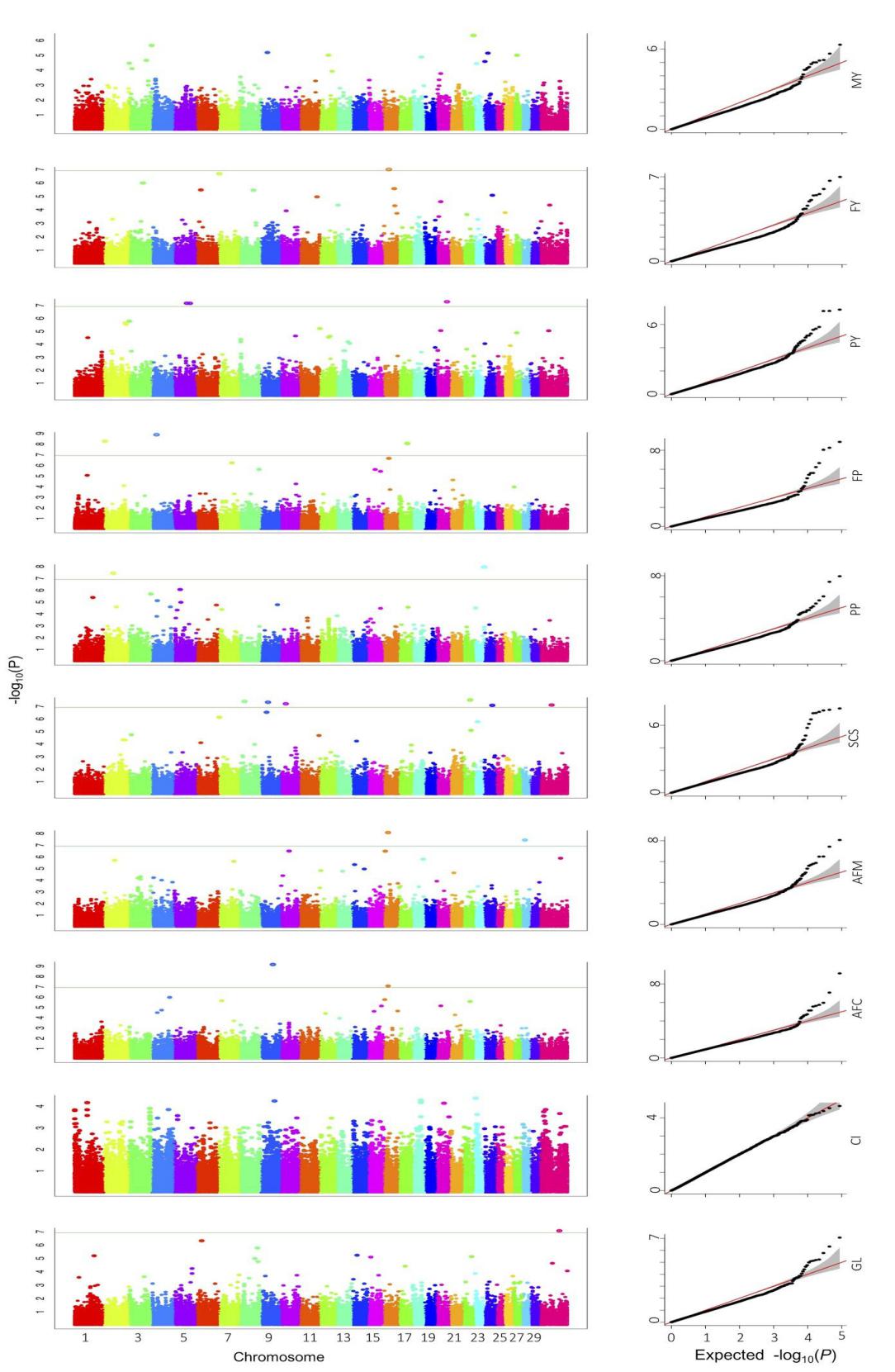
**

Supplement: Supplementary file 6 — Additional file 6: Figure S6. Manhattan and Q-Q plots of GWAS results, without considering population structure. These Manhattan plots (left) illustrate results from an association analysis model that did not consider population structure. GWAS was performed with FarmCPU software and a significant p-value threshold set at P = 10–7. Q-Q plots (right) are displayed as scatter plots of true and expected log p-values. Milk traits include milk yield (MY), fat yield (FY), protein yield (PY), fat percentage (FP), protein percentage (PP), and somatic cell score (SCS). Reproductive traits include age at first service (AFS), age at first calving (AFC), gestation length (GL), and calving interval (CI). [file 12864_2019_6224_MOESM6_ESM.docx]
