# Supplementary material for: Genome-wide association study of milk and reproductive traits in dual-purpose Xinjiang Brown cattle
Source: BMC Genomics. 2019 Nov 8;20:827. doi: 10.1186/s12864-019-6224-x (PMC6842163; doi:10.1186/s12864-019-6224-x)
Supplement: Supplementary file 7 — Additional file 7: Table S1. Genomic inflation factor (lambda) of each trait. [file 12864_2019_6224_MOESM7_ESM.docx]

**Table S1. Genomic inflation factor (lambda) by traits.**

| Trait | Lambda Without PC | Lambda With PC |
| --- | --- | --- |
| Milk yield (MY) | 0.91 | 0.88 |
| Fat yield (FY) | 0.95 | 0.95 |
| Protein yield (PY) | 0.93 | 0.91 |
| Fat percentage (FP) | 0.93 | 0.85 |
| Protein percentage (PP) | 0.91 | 0.90 |
| Somatic cell score (SCS) | 1.02 | 0.99 |
| Age at first service (AFS) | 0.90 | 0.90 |
| Age at first calving (AFC) | 0.88 | 0.88 |
| Gestation length (GL) | 0.98 | 0.94 |
| Calving interval (CI) | 0.83 | 0.83 |
